# Supplementary material for: Differential Regulation of Hyaluronan Synthesis by Three Isoforms of Hyaluronan Synthases in Mammalian Cells
Source: Biomolecules. 2024 Dec 9;14(12):1567. doi: 10.3390/biom14121567 (PMC11673962; doi:10.3390/biom14121567)
Supplement: Supplementary file 1 [file biomolecules-14-01567-s001.zip › biomolecules-3309181-supplementary.pdf]

Supplementary Materials

Differential Regulation of Hyaluronan Synthesis by Three Isoforms of Hyaluronan Synthases in Mammalian Cells

Jie Wang [1,2], Zhikun Wu [1,2], Longtao Cao [1,2] and Feng Long [1,2,*]

[1] Department of Neurosurgery, Zhongnan Hospital of Wuhan University, School of Pharmaceutical Sciences, Wuhan University, Wuhan 430071, China

[2] Ministry of Education Key Laboratory of Combinatorial Biosynthesis and Drug Discovery, School of Pharmaceutical Sciences, Wuhan University, Wuhan 430071, China

[*] Correspondence: longfe@whu.edu.cn

This supplementary document includes:

- Supplementary Methods
- Supplementary Figures S1–S4

Supplementary Methods

Cell Culture

HEK293T cells (Gibco) were cultured in Dulbecco's modified Eagle Medium (DMEM, containing 4.5 mg/mL glucose, Gibco) supplemented with 10% fetal bovine serum (FBS, HyClone, Thermo Scientific), 4 mM glutamine (Sigma), and 50 g/mL streptomycin sulfate and 50 units/mL penicillin (Sigma). Cells were passaged three times a week at a 1:5 split ratio using 0.05% trypsin (w/v) 0.02% EDTA (w/v) (Biochrom AG, Berlin, Germany).

Transfections of human HAS1, HAS2 and HAS3 in HEK293T cells

DNA fragments encoding human HAS1, HAS2 and HAS3 were fused to a C-terminal eGFP tag using conventional PCR cloning techniques. These constructs were cloned into the pEGBacMam plasmid for constitutive expression under the control of the CMV promoter. HEK293T cells were seeded on 24-well plates (Corning) for biochemical analyses. The next day, the cells were transfected with FuGENE 6 transfection reagent (Promega).

Western blotting

For western blot experiments, PVDF membranes (Bio-rad) were activated for 5 min in 100% methanol and incubated for 5 min in the transfer buffer (1× Tris-glycine, 20% methanol). A wet transfer was performed for 120 min at 300 mA in a Bio-rad Trans-Blot. The membranes were blocked with 5% non-fat dry milk in 1 X TBST and incubated for 2 h at room temperature. Primary antibodies were added at dilutions of 1:3000 Anti-His tag (mouse, GenScript) and 1:3000 Anti-Flag tag (rabbit, GenScript), and incubated overnight at 4 °C. Membranes were washed 6 times with TBST and incubated with corresponding secondary antibodies at room temperature for 1h. After incubation with the secondary antibodies, membranes were treated with ECL reagent and exposed in Bio-rad’s multi-application gel imaging system for

protein detection.

Bioinformatics Analysis of HAS1, HAS2, and HAS3

GeneBank accession numbers are as follows: HsHAS1, AAF87845.1; MmHAS1, NP_032241.1; XIHAS1, NP_001079696.1; OmHAS1, XP_067094336.1; BtHAS1, XP_024834545.1; OaHAS1, XP_027833278.2; FhHAS1, XP_012710979.1; TaHAS1, XP_052512802.1; LvHAS1, XP_007463459.1; HsHAS2, NP_005319.1; MmHAS2, NP_032242.3; XIHAS2, NP_001083837; OmHAS2, XP_067094317.1; BtHAS2, NP_776504.2; OaHAS2, XP_004011715.1; FhHAS2, XP_035985981.1; TaHAS2, XP_052507764.1; LvHAS2, XP_007445648.1; HsHAS3, AAK73797.1; MmHAS3, NP_001317977.1; XIHAS3, XP_041417114.1; OmHAS3, XP_067091357.1; BtHAS3, NP_001179796.1; FhHAS3, XP_012707764.2; TaHAS3, XP_044788036.2; SeHAS, WP_012514859.1; CvHAS, AGE52748.1; SpHAS, WP_011018340.1; SzHAS, HEK9535426.1; SuHAS, CAB46918.2.

Kinetic properties analysis

Kinetic analyses were performed by varying the concentration of one substrate while maintaining the other at a saturating level. Real-time quantification of UDP release was achieved using an enzyme-coupled reaction that oxidizes NADH. During the process of HA chain extension, HASs transfer GlcA and GlcNAc from UDP-GlcA and UDP-GlcNAc to the reducing end of HA, releasing UDP as a byproduct. The released UDP reacted with phosphoenolpyruvate (PEP) in the presence of pyruvate kinase, producing uridine triphosphate (UTP) and pyruvate. Subsequently, the product pyruvate was reduced to L-lactate by L-lactate dehydrogenase (L-LDH), which simultaneously facilitated the oxidation of NADH to NAD^+ (Fig. S2). NADH absorbs moderately at 340 nm (ε = 5740 M^{-1} cm^{-1}) compared with NAD^+ (ε = 7.8 M^{-1} cm^{-1}), thus providing the basis for a signal decrease assay for HAS.

$$\text{n UDP-GlcA} + \text{n UDP-GlcNAc} + \text{HA} \xrightarrow{\text{HASs}} \text{(GlcA-GlcNAc)n-HA} + \text{2n UDP} \quad (1)$$

$$\text{UDP} + \text{PEP} \xrightarrow{\text{Pyruvate kinase}} \text{UTP} + \text{pyruvate} \quad (2)$$

$$\text{Pyruvate} + \text{NADH} \xrightarrow{\text{L-LDH}} \text{L-Lactate} + \text{NAD}^{+} \quad (3)$$

Figure S1. Schematic representation of the enzyme-coupled assay for HAS1, HAS2 and HAS3. HASs transfers GlcA and GlcNAc from UDP-GlcA and UDP-GlcNAc to the reducing end of HA in order to extend the HA chain. The assay depends on coupling the release of UDP generated from Eq. (1) to PEP in the presence of pyruvate kinase (Eq. (2)). Then pyruvate of Eq. (2) is reduced to L-lactate with concomitant oxidation of NADH into NAD+ (Eq. (3)). The reduction in NADH absorbance is continuously monitored.

Figure 3A

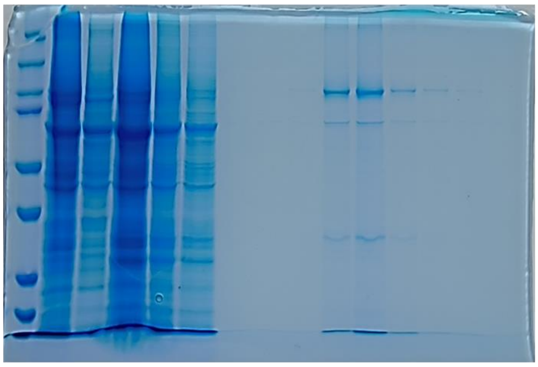

Figure 3B

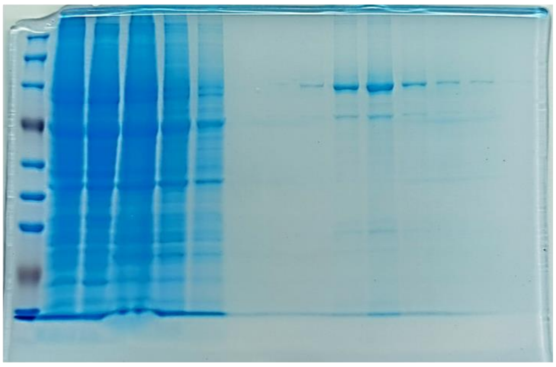

Figure 3C

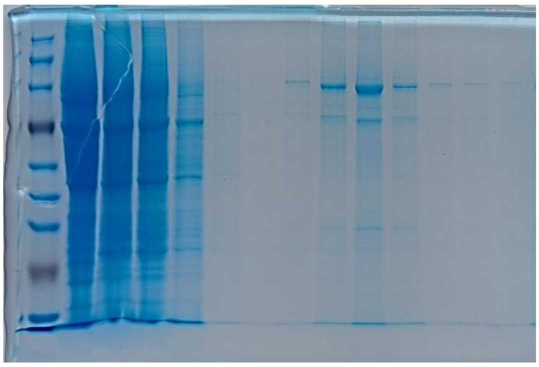

Figure S2. Original gel images for Figure 3A, 3B, and 3C.

Figure 3D

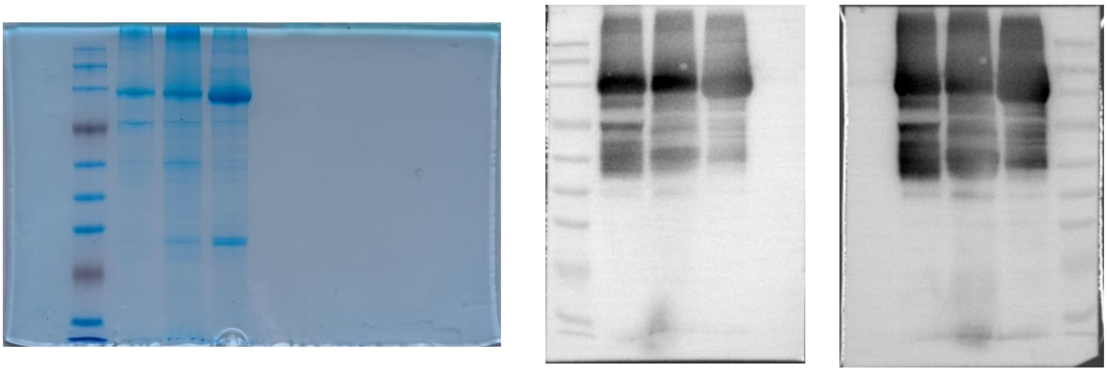

Figure S3. Original gel images for Figure 3D.

Figure 4A

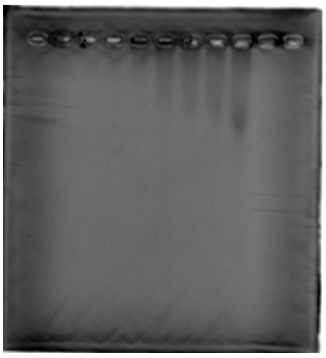

Figure 4B

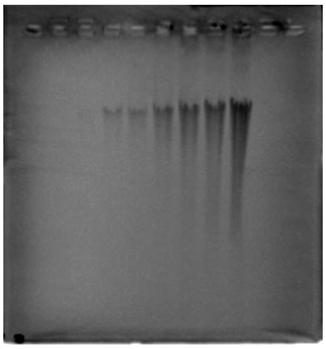

Figure 4C

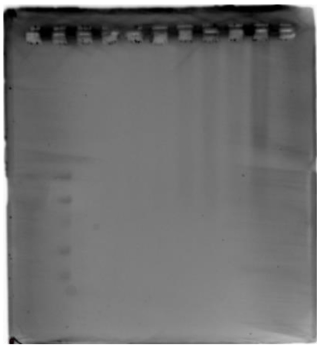

Figure 4D

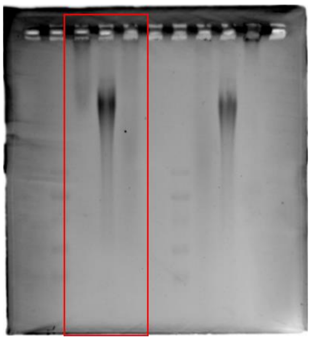

Figure 4E

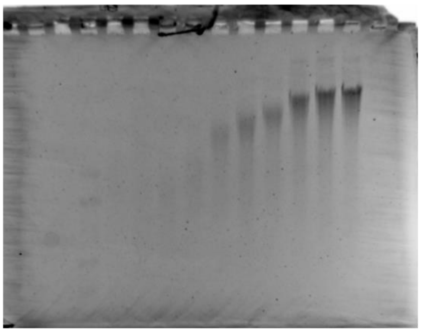

Figure 4F

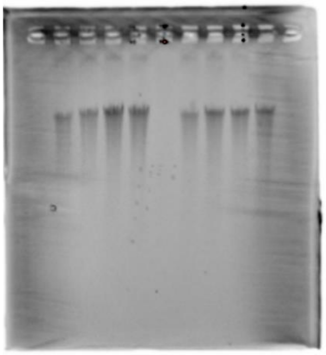

Figure S4. Original gel images for Figure 4A-4F.
